# Influence of Rivastigmine transdermal on butyrylcholinesterase levels in patients with Alzheimer's disease

**DOI:** 10.1590/S1980-57642011DN05040012

**Published:** 2011

**Authors:** Gustavo Alves Andrade dos Santos, Paulo Renato Canineu, Ivair Donizette Gonçalves, Paulo Celso Pardi

**Affiliations:** 1 Professional Master of Pharmacy from Uniban Brasil, São Paulo SP, Brazil; 2 Faculty Member of the Pontifical Catholic University of São Paulo (PUC), São Paulo SP, Brazil

**Keywords:** Alzheimer's disease, rivastigmine, acetylcholine, Mini Mental State Examination, Neuropsychiatric Inventory, esterases

## Abstract

Cholinesterase inhibitors (ChE-Is) are among the main drugs approved for the treatment of Alzheimer's disease (AD). Rivastigmine in the form of a transdermal patch is an alternative delivery method, and can give greater treatment compliance.

**Objectives:**

To conduct a preliminary assessment of the neurocognitive and biological effects of oral and transdermal Rivastigmine in patients with AD and to identify a potential biological marker and demonstrate a possible relationship between esterase levels and behavioral scores of AD patients.

**Methods:**

Forty patients with AD were treated with cholinesterase inhibitors (ChE-Is), evaluated using the MMSE and NPI, and simultaneously sampled to determine their serum levels of AChE and BuChE for 180 days.

**Results:**

The differences obtained between oral and transdermal forms, as assessed by the MMSE and NPI scores of the AD patients, were not significant at the three time points examined (0, 90, and 180 days). However, serum BuChE levels of the transdermal group differed significantly (p<0.0004) compared with those of the oral group at 90 days.

**Conclusion:**

Use of a transdermal ChE-I, rivastigmine tartrate significantly reduced BuChE levels in the AD patients studied.

## Abstract (pt)

Os inibidores das colinesterases estão entre as principais drogas aprovadas para tratamento da doença de Alzheimer (DA). Rivastigmina na forma de adesivo transdérmico é um método alternativo de liberação e pode fornecer uma maior aderância ao tratamento.

**Objetivos:**

Conduzir uma abordagem preliminar dos efeitos neurocognitivos e biológicos da rivastigmina oral e transdérmica em pacientes com DA e identificar um potencial marcador biológico e demonstrar uma possível relação entre níveis de esterases e escores de comportamento de pacientes com DA.

**Métodos:**

Quarenta pacientes com DA com inibidores de colinesterases foram avaliados usando o MEEM e o INP e colhidas amostras para determinar seus níveis séricos de AChE e BuChE por 180 dias.

**Resultados:**

As diferenças obtidas entre as formas oral e transdérmica, avaliadas pelo MEEM e INP não diferiram em três ocasiões (0, 90 e 180 dias). Todavia, os níveis de BuChE no grupo transdérmico diferiu significativamente (p<0.0004) comparados ao grupo de administração oral em 90 dias.

**Conclusão:**

O uso do tartarato de rivastigmina, forma transdérmica reduziu significativamente os níveis de BuChE nos pacientes estudados com DA.

## Introduction

The development of Alzheimer's disease (AD) is marked by a gradual or progressive deterioration of intellectual function, significant decline in the ability to perform everyday activities, and changes in personality and behavior, resulting in impaired memory, attention, executive function, language, and ability to perform calculations and abstractions. Personality changes are frequent, with patients becoming more passive or aggressive and less spontaneous.^1,2^

Cholinesterase inhibitors (ChE-Is) are among the main drugs approved for the treatment of AD. Their use is based on the assumption that cholinergic deficits occur during the disease and the inhibitors function by increasing the availability of synaptic acetylcholine (ACh) by inhibiting its key catalytic enzymes, acetylcholinesterase (AChE) and butyrylcholinesterase (BuChE).^3,4^ Currently, ChE-Is represent the most promising therapeutic agents and are the only therapeutic class of drugs developed that have produced significant cognitive improvement in AD patients.^5^ In 2003, Trinh et al.^6^ stated that studies examining AD treatments have focused on reducing cognitive decline by using ChE-Is. Cummings endorsed ChE-Is as the first class of drugs currently used for this purpose.^7^ In addition to ChE-Is, there are non-competitive glutamate receptor antagonists (N-methyl d-aspartate), such as memantine, which block the pathological effects of high glutamate levels^8^ and were the first of a novel class of drugs designed to treat moderate to severe AD. The combination of ChE-Is with NMDA receptor antagonists in the treatment of AD may result in improved results compared with non-pharmacological therapies. However, the potential adjuvant effects of suppressing auxiliary psychotropic drug therapy have yet to be addressed, although it is known that rivastigmine, one of the ChE-Is, reduces or eliminates the need to take these other drugs. Rivastigmine is a well-tolerated drug that improves cognition and participation in activities of daily living among patients at mild to moderately severe stages of AD.^9^ Furthermore, in 1998, it became the first approved ChE-I to be sold in Brazil. It is one of the most widely used drugs for the treatment of AD because it is capable of inhibiting both AChE and BuChE and, consequently, is more effective at increasing brain levels of ACh.^4^ Rivastigmine in the form of a transdermal patch is the preferred delivery method by caregivers of AD patients because it ensures greater treatment compliance.^10^ This ChE-I represents, from a clinical perspective, an effective treatment option for people with AD.^11^

Forlenza showed that second-generation ChE-Is (i.e., rivastigmine, donepezil, and galantamine) have the same pharmacological properties, and similar side effects (nausea, vomiting, diarrhea, increased acid secretion, dyspepsia, anorexia, and abdominal pain).^3^

The aim of this study was to evaluate the neurocognitive and biological effects of administering oral and transdermal rivastigmine tartrate to individuals with dementia associated with AD. The study also sought to identify a possible marker for assessing this treatment outcome using biochemical results and behavioral and cognitive evaluations of AD patients. Hence, a possible relationship between pre-diagnostic blood levels of AChE and BuChE and cognitive and behavioral scores of the AD patients was demonstrated. Therefore, to investigate the influence of rivastigmine tartrate using the aforementioned evaluations, the effectiveness of various formulations of rivastigmine in the pharmacological treatment of patients with AD was assessed.

The biological quantification of these substances, in addition to their activities, allow establishment of parameters to determine the rivastigmine levels at which neuropsychiatric symptoms associated with this disease are reduced.

## Methods

A total of 40 individuals of both gender with mild- to moderate-stage AD, diagnosed at the beginning of the trial, were studied. Patients were grouped according to type of rivastigmine tartrate treatment where 20 patients were assigned to the oral group (OG) and 20 patients to the patch group (PG). Subsequently, neurocognitive surveys and biological blood analyses were performed over a period of 180 days. The rating determined on the Neuropsychiatric Inventory (NPI) enabled the tracking of the effectiveness of the treatments, and the severity of the disease was classified as either mild, moderate, or severe.^12^ Inclusion and exclusion criteria were defined based on a script from the National Institute of Neurological and Communicative Disorders and Stroke (NINCDS) and the Alzheimer's Disease and Related Disorders Association (ADRDA, 2008). The instruments used to assess neurocognition during the experiment were the Mini-Mental State Examination (MMSE) and the NPI. AChE and BuChE were analyzed according to the Accreditation Program for Clinical Laboratories (PALC) standards from the Brazilian Society of Clinical Analyses (SBAC). The study was approved by the Ethics and Research Committee of the UNIBAN BRASIL (protocol no. 292/08). For statistical analyses, ANOVA, Student's t-test, and the Kruskal-Wallis test were employed on GraphPad Prism 5 software.

## Results

### Neurocognitive evaluation

**Evaluation of Mini-Mental State Examination (MMSE) and Neuropsychiatric Inventory (NPI) results at start of treatment** - The patients were clinically evaluated using the NINCDS inclusion and exclusion criteria, and grouped according to form of treatment. The OG comprised patients treated with a 6.0-mg dose of rivastigmine tartrate every 12 hours, whereas the PG comprised patients treated with a transdermal patch containing a 9.5-mg dose of rivastigmine tartrate every 24 hours. Preliminary analysis of patients' clinical status revealed similar neurocognitive results. The patients exhibited no significant differences in MMSE scores (p=0.30). There were also no significant differences in NPI scores between the groups (p=0.43). Thus, results confirmed patients had identical neurocognitive function at the initiation of treatment with no significant differences found between the two groups.

On the MMSE assessment at 180 days, the OG had significantly lower scores compared to day 0. Nevertheless, the MMSE for the PG in the same period exhibited an insignificant decrease compared with day 0 (Table 1). On the NPI assessment at 180 days, the OG had significantly lower scores compared with day 0. However, in the same period, the PG exhibited a significant decrease compared with day 0 (Table 2).

**Table 1:** Mini-Mental State Examination evaluation at two time points.

	Day 0 (zero)	Day 180
Oral	19.4±4.1	14.1±7.0*
Patch	20.2±7.0	16.2±6.8**

* p<0.0002, decrease compared to day-0 group (paired t-test);

** p<0.0006, decrease compared to day-0 group (paired t-test).

**Table 2:** Neuropsychiatric inventory evaluation at two time points.

	Day 0	Day 180
Oral	33.4±11.2	27.1±12.3*
Patch	40.4±20.2	29.3±18.0**

* p<0.01, decrease compared to day-0 group (paired t-test);

** p<0.001, decrease compared to day-0 group (paired t-test).

### Biochemical assessment

***AChE levels*** - The results of biochemical measurements of AChE levels in the OG and PG patients (Table 3) revealed no significant changes (p>0.05) from 0 to 180 days across the three time periods examined.

**Table 3:** Evaluation of AChE levels at three time points

	0 dd	90 dd	180 dd
Oral	3.20±0.58	2.99±0.87	3.39±0.69
Patch	3.25±0.62	3.31±0.52	3.47±0.49

Comparison of AChE levels after 90 days of rivastigmine tartrate treatment revealed a slight change in AChE levels in the PG although this difference was not statistically significant (p=0.1764; non-parametric t-test).

***BuChE levels*** - The results of biochemical measurements of BuChE levels of the OG and PG patients (Table 4) revealed altered levels after 180 days of treatment when comparing scores at day 0 using ANOVA. A significant difference between the OG and PG patients was observed at both experimental days 0 and 90. However, the same difference between the groups was not observed after 180 days.

**Table 4:** Evaluation of BuChE levels at three time points (values in U/L).

	0dd	90dd	180dd
Oral	4179.5±1799.2	3782.9±1798.1	5544.6±2109.5*
Patch	6618.2±2095.6**	6165.5±2090.5***	6339.4±2451.1

* p<0.05, compared with oral group at day 90 (ANOVA test);

** p<0.003, compared with oral group at day 0 (non-parametric t-test);

*** p<0.0004, compared with oral group at day 90 (non-parametric t-test).

## Discussion

Our results showed that, at day 0, the MMSE scores of the OG and PG were the same (Table 1), with a score of 19.4 for the OG versus 20.2 for the PG.

In 2007, the IDEAL^13^ study described the MMSE as an assessment tool for evaluating AD (scores from 10-20).

These values are similar to those previously described by Almeida and Crocco, who analyzed a group of institutionalized elderly in the Santa Casa de São Paulo Hospital. The patients in the cited study had a mean MMSE score of 14.93 (CI, 12.68 to 17.18).^14^

In 2003, Laks et al. found an MMSE score of 22.34 in literate and 17.08 in illiterate individuals.^15^ These data reinforce the correlation between schooling and MMSE performance.^16^ Some studies have suggested setting a score of 17 as the MMSE cutoff point for individuals with low education.^17^ Similarly, Almeida suggested 20 as the optimum cutoff score for diagnosing AD in elderly individuals with no schooling.^18^

From these evaluations, we can conclude that schooling has a substantial impact on cognitive performance, as assessed by the MMSE. However, if stratification had been applied in these populations, it would have been possible to clarify any possible interference of schooling.

The MMSE is a screening tool, and it has been suggested that individuals with low scores and possible functional losses undergo more detailed neuropsychological evaluation.^19^

In general, studies have shown that the use of rivastigmine has been beneficial for patients with AD. These studies have emphasized the improvement of both cognition and global performance.^10,20,21^

We believe that this significant improvement contributes to stabilization of the patient's state for several months. However, no beneficial effects persist at more advanced stages of the disease.

In Table 2, results of NPI assessments at the beginning of treatment show that OG patients had a score of 33.4 and PG patients a score of 40.4. This indicates a balanced behavioral situation.

The NPI is used to detect and quantify changes arising from psychiatric disorders caused by dementia.^22^ The NPI consists of an interview designed to assess ten behavioral areas: delusions, hallucinations, agitation, dysphoria, anxiety, apathy, irritability, euphoria, disinhibition, and aberrant motor behavior. Two other areas may be studied in this assessment: nighttime behavioral disturbances and appetite and eating abnormalities.^23^ Based on the scores obtained, a possible correlation was evident between results on the neurocognitive assessment and AChE and BuChE levels, as described below. For the groups selected for this research, differences between oral and patch treatments were not significant after 180 days. However, the lower NPI scores of PG patients compared with OG patients is associated with a major decrease in their behavioral assessment. Scores were 27.1 in the OG versus 29.3 for the PG - a difference not reaching statistical significance.

Importantly, in this study we found a relationship between AD and inflammation, and also identified AChE and BuChE as possible markers for low-grade inflammation.^20^ AChE is found at high levels in the brain, nerves, and red blood cells, whereas BuChE (pseudocholinesterase) is found in the blood, pancreas, liver, and central nervous system (CNS).^24,25^ In a study by Giacobini et al., AChE was found at cholinergic nerve terminals, whereas BuChE was associated with glial cells or neurons.^26^ Clearly establishing the role of BuChE in the normal brain, or in brains with AD, remains a challenge.^27^ The biochemical assessment of blood from patients with AD conducted in the present work confirmed the importance of ChE-Is as a potential treatment strategy, since ChE-Is were shown to influence serum esterase levels of patients with AD, enabling monitoring of the disease by measuring concentrations of these enzymes. No significant changes in blood AChE level were observed from day 0 (start of sample collection) to day 180 (final sampling) with its value remaining stable. In patients treated with the oral form of the drug for six months, differences showed a p-value of >0.05, while blood AChE levels in OG patients exhibited a slight decrease during the latter 90 days of treatment (between day 90 and day 180). In addition, for the patch form of drug treatment, the samples showed differences yielding a p-value of >0.05, with a slight decrease at treatment day 180 compared with day 0.

BuChE levels (Table 4) of the OG and PG differed significantly at day 0. Similarly, patients who used the patch form of the drug differed (p>0.0004) to patients using the oral form. The mean BuChE level of patients who used the oral form was 4,179.5 U/L, compared to 6,618.6 U/L in patients who used the patch form. During the trial, those patients who used the patch form for 90 days continued to exhibit a significant difference (p>0.003) while at study endpoint, both groups were statistically similar. This outcome may reflect the inhibition of rivastigmine tartrate, which strongly influences BuChE levels. The findings of this study confirm the pharmacological effects of rivastigmine on esterases, specifically as an inhibitor of BuChE, where its effects were observed by sample analysis.

These results are of interest since no previous studies have found a correlation between biochemical and behavioral data. The small changes observed in MMSE and NPI scores indicate no significant cognitive differences.

Fluctuations were significant on the biochemical BuChE analysis, indicating that BuChE levels can be monitored using this test and may serve as a parameter for measuring disease evolution and treatment and also facilitate clinical monitoring of patients with AD.

Although the results of this study suggest that biomarkers can be used as potential AD diagnostic tools, as previously described in the literature,^28^ several limitations were found in connection with the study. Firstly, the sample size of 40 patients limits the observational scope of the effects of rivastigmine. In addition, we believe that our cholinesterase determination method may not have accurately quantified the concentrations of these substances in the evaluated patients. However, in this study, these limitations were not believed to have interfered with any of the other drugs used by the patients.

Therefore, further studies are necessary to evaluate the use of biological markers as monitoring and evaluation tools. In conclusion, the transdermal form of rivastigmine (compared to the oral form) showed a significant difference in its reduction of BuChE, confirming its ability to inhibit this enzyme which is typically elevated in the advanced stages of AD. In addition, the use of patch technology yields similar or better results than oral administration, offering patients greater convenience.
